# Population differentiation of zander (*Sander lucioperca*) across native and newly colonized ranges suggests increasing admixture in the course of an invasion

**DOI:** 10.1111/eva.12155

**Published:** 2014-04-26

**Authors:** Erik Eschbach, Arne W Nolte, Klaus Kohlmann, Petra Kersten, Jochem Kail, Robert Arlinghaus

**Affiliations:** 1 Department of Biology and Ecology of Fishes, Leibniz-Institute of Freshwater Ecology and Inland FisheriesBerlin, Germany; 2 Max Planck Institute for Evolutionary BiologyPlön, Germany; 3 Department of Ecophysiology and Aquaculture, Leibniz-Institute of Freshwater Ecology and Inland FisheriesBerlin, Germany; 4 Department of Aquatic Ecology, Faculty of Biology, University of Duisburg-EssenEssen, Germany; 5 Chair of Integrative Fisheries Management, Faculty of Agriculture and Horticulture, Humboldt-Universität zu BerlinBerlin, Germany

**Keywords:** competitive exclusion, gene flow, hybridization, local adaptation, pike-perch, secondary contact

## Abstract

In addition to ecological factors, evolutionary processes can determine the invasion success of a species. In particular, genetic admixture has the potential to induce rapid evolutionary change, which can result from natural or human-assisted secondary contact between differentiated populations. We studied the recent range expansion of zander in Germany focusing on the interplay between invasion and genetic admixture. Historically, the rivers Elbe and Danube harboured the most north-western source populations from which a north-westward range expansion occurred. This was initiated by introducing zander outside its native range into rivers and lakes, and was fostered by migration through artificial canals and stocking from various sources. We analysed zander populations of the native and invaded ranges using nuclear and mitochondrial genetic markers. Three genetic lineages were identified, which were traced to ancestral ranges. Increased genetic diversity and admixture in the invaded region highlighted asymmetric gene flow towards this area. We suppose that the adaptive potential of the invading populations was promoted by genetic admixture, whereas competitive exclusion in the native areas provided a buffer against introgression by novel genotypes. These explanations would be in line with evidence that hybridization can drive evolutionary change under conditions when new niches can be exploited.

## Introduction

Contemporary environmental alterations are predominantly caused by human activity. As a result, populations may be reduced or increased in numbers, and the species as a whole may contract or expand its range. This, in turn, can lead to changes in species composition and biodiversity, particularly in invaded areas (Walther et al. 2002; Willis and Bhagwat 2009). Anthropogenic causes of biological invasions are diverse. Together with the modification of ecosystems, they include accidental (Karatayev et al. 2012; Bacela-Spychalska et al. 2013; Papini et al. 2013) as well as intentional mass releases of species, particularly in agriculture, aquaculture and via recreational activities. Through both pathways, many organisms have been distributed far beyond their natural barriers (Kolar and Lodge 2001; Laikre et al. 2010; Crispo et al. 2011).

However, biological invasions are not only anthropogenic. They have often occurred in the past in the course of climatic changes, like the succession of cold and warm periods. But in contrast to these slow processes, human-induced changes are much faster. A stable ecosystem with an intact biological community might withstand the influence of invaders and maintain its functionality and genetic identity, but disturbed systems may not (Englbrecht et al. 2002; Hansen 2002; Larsen et al. 2005). Establishment and dispersal is, furthermore, influenced by whether a niche is occupied or not, and how competitive the particular species or population is relative to the invader. It is far easier for an invader to establish and spread if the competitive environment is less hostile or if an invading species is competitively superior compared with the resident species (Waters et al. 2013). The sheer number of individuals released annually into the wild is another important factor that drives the establishment of invaders by swamping a place and outcompeting existing species. Generally, high propagule pressure provides an invading species or population with a time credit allowing it to establish in the new habitat even if initial establishments fail (Green 1997; Kolar and Lodge 2001; Lambrinos 2004; Waters et al. 2013).

In addition to exogenous factors that affect the success of a biological invasion, there is also a possible endogenous contribution of the invading organism itself. Successful invasive species typically exhibit a broad environmental plasticity and are often migratory. However, species that are ecologically less flexible can also become successful invaders, if the newly colonized area provides similar environmental conditions as the invaders' ancestral area (Kolar and Lodge 2001) or if the organism evolves on short time periods to become a better invader (Keller and Taylor 2010; Krehenwinkel and Tautz 2013). Irrespective of the nature of the environmental changes that an organism is faced with, the colonization of a new range is most likely successful when organisms adapt quickly to altered environmental conditions. Admixture of genetic lineages with different genetic backgrounds can be considered as a particularly extreme case of recruitment of ancestral genetic variance and is thought to contribute to evolutionary change. The central idea is that new combinations of alleles from previously isolated gene pools may facilitate adaptation to a new environment (Nolte and Tautz 2010; Abbott et al. 2013). There is growing evidence that hybridization and the process of admixture that follows can indeed be potent drivers of fast adaptation rather than being deleterious in invasive animals and plants (Kolbe et al. 2004; Keller and Taylor 2010; Krehenwinkel and Tautz 2013). In fishes, cichlids *Cichlidae* (Schliewen and Klee 2004), sharpfin silversides *Telmatherinidae* (Herder et al. 2006) and whitefish *Coregonidae* (Bernatchez 2004; Hudson et al. 2011) have experienced genetic admixture in the past correlated with evolutionary changes. A tight correlation of recent admixture and the invasion of perturbed habitats has been observed for sculpins *Cottus* spp. (Nolte et al. 2005; Stemshorn et al. 2011). Analyses of hybrid zones of invasive sculpins with noninvasive sculpins by Nolte et al. (2006) demonstrated an increased fitness of admixed invaders in a new habitat, while niches already occupied by locally adapted sculpins are not easily taken over by invaders. Lucek et al. (2010) found that between-population genetic differentiation of at least three genetic lineages was converted into within-population variation in three-spined sticklebacks *Gasterosteus aculeatus* that had recently invaded the midlands of Switzerland. Hence, under particular conditions hybridization may elevate fitness and contribute to the invasion success in areas where no adapted populations exist. In contrast, hybridization usually reduces fitness in already established populations, providing a buffer against introgression of new genes (Araki et al. 2007; Fraser et al. 2011).

Here, we used zander *Sander lucioperca*, Linnaeus, 1758 as a model species to study the role of genetic admixture in an invasion process. After the last ice age, this species started its spread from the Caspian-Black Sea region reaching the Baltic Sea during the Lake Ancylus period approx. 6000 years ago (Lönnberg 1898; Reid and Orlova 2002; Haponski and Stepien 2013). In Germany, the Elbe and Danube drainages represented the north-western most European dispersal limits, until the late 19th century when zander was introduced by humans into rivers beyond these natural frontiers (Welcomme 1988; Löffler 1998; Larsen and Berg 2004; Kottelat and Freyhof 2007 (listing zander explicitly as an invasive species). As a commercially valuable species, zander is still regularly stocked in both its native and non-native ranges. Canals connecting ancestral and novel drainages have further facilitated the dispersal and provided multiple opportunities for secondary contacts of zander populations (e.g. through the Elbe-Weser-Schifffahrtsweg, Mittellandkanal and Rhein-Main-Donau Kanal connecting different catchments in Germany). Moreover, zander is euryoecious, that is, it is able to cope with a broad range of environmental conditions including low salinity brackish waters (Winkler et al. 1989; Lehtonen et al. 1996; Khurshut and Kohlmann 2009), which increases its potential for dispersal and invasion (Poulet et al. 2009).

The objective of our study was to trace the origins and population structure of zander across central European waters in Germany. Despite their broad tolerance and dispersal capacity, zander populations in their native catchments should be evolutionarily adapted to their environments. Consequently, allochtonous zander populations should have difficulties establishing in these areas due to competitive exclusion (Waters 2011), similar to other fishes in comparable situations (Englbrecht et al. 2002; Hansen 2002; Larsen et al. 2005). In contrast, the lack of established conspecific competitor populations in the areas outside the native range of zander might have provided the chance for genetic admixture enhancing adaptive potential (Nolte and Tautz 2010). If this applies, we expect to see lower levels of admixture in native ranges and higher levels of admixture in the invasive range.

## Materials and methods

### Sampling and DNA extraction

Thousand four hundred and seventy-eight fin and muscle tissue samples of zander were collected from rivers, lakes and canals in Germany by commercial and recreational fishers, research organizations and state fishery authorities. Samples were collected from seven main river catchments including different numbers of tributaries and lakes, with more than one sampling site at some of the larger water bodies (Table 1). For most of these sites, 10–30 samples were obtained as frozen tissues. After short-term storage at −20°C, samples were thawed in absolute ethanol (Thomas Geyer, Renningen, Germany) at room temperature and subsequently transferred to fresh ethanol following Eschbach (2012). Samples from research organizations were obtained preserved in ethanol, while samples from anglers were obtained air-dried. DNA was successfully extracted from 1163 samples with the PeqGOLD Tissue DNA Mini Kit (Peqlab Biotechnologie GmbH, Erlangen, Germany) according to the manufacturer's instruction.

**Table 1:** Sampled water bodies and geographic position of sampling sites in Germany. Sample identification (ID) is given by a three letter code, which is used throughout the text

Catchment: names & types of waterbody		Sample: ID & size		Geographic position: latitude north, longitude east & German federal state ID		
Danube						
Danube	r	DON	5	48°33′N	13°37′E	BY
Ilz	r	DON	4	48°35′N	13°27′E	BY
Inn	r	DON	7	48°17′N	13°09′E	BY
Lech	r	DON	1	48°20′N	10°56′E	BY
Regen	r	DON	4	49°11′N	12°17′E	BY
Rott	r	DON	16	48°23′N	12°36′E	BY
Schwarzach	r	DON	1	49°24′N	12°12′E	BY
Wertach	r	DON	3	47°36′N	10°26′E	BY
Chiemsee	l	CHS	27	47°52′N	12°27′E	BY
Waginger See	l	WAS	15	47°57′N	12°44′E	BY
Ammersee	l	AMS	29	48°00′N	11°07′E	BY
Starnberger See	l	STS	5	47°53′N	11°18′E	BY
Elbe						
Elbe	r	ELB1	23	53°04′N	11°27′E	BB
		ELB2	26	53°22′N	10°32′E	NI
		ELB3	27	53°23′N	10°13′E	NI
		ELB4	32	53°01′N	11°27′E	NI
		ELB9	30	51°51′N	12°27′E	SN
Nord-Ostsee-Kanal	c	NOK1	18	54°20′N	09°44′E	SH
		NOK3	8	54°03′N	09°18′E	SH
		NOK4	10	54°20′N	09°45′E	SH
Haaler Au	r	HAA	26	54°11′N	09°30′E	SH
Windebyer Noor	l	WIN	29	54°28′N	09°48′E	SH
Havel	r	HAV1	31	52°25′N	12°33′E	BB
		HAV2	29	52°44′N	12°15′E	BB
Müggelsee	l	MUE	52	52°26′N	13°39′E	BB
Großer Kossenblatter See	l	GKB	31	52°09′N	14°13′E	BB
Müritz	l	MUR	30	53°25′N	12°41′E	MV
Plauer See	l	PLS	20	53°25′N	12°41′E	MV
Oder						
Oder	r	ODE3	30	52°41′N	14°25′E	BB
		ODE4	22	52°16′N	14°35′E	BB
Peene Haff	e	PEH	27	53°51′N	13°49′E	MV
Stetiner Haff	e	STH	33	53°45′N	14°04′E	MV
Strelasund	Baltic Sea	STR	20	54°16′N	13°07′E	MV
Rhine						
Rhine	r	RHE1	22	48°51′N	08°06′E	BW
		RHE2	31	51°45′N	06°20′E	NW
Altrhein	r	RHE3	24	49°07′N	08°21′E	BW
		RHE4	31	49°09′N	08°22′E	BW
Mosel	r	MOS	35	49°47′N	06°49′E	RP
Main	r	MAI3	10	49°49′N	09°52′E	BY
		MAI4	16	50°00′N	09°03′E	BY
Lake Constance	l	BOS2	31	47°32′N	09°36′E	BW
		BOS3	25	47°35′N	09°31′E	BW
Weser						
Edersee	l	EDS	31	51°11′N	09°03′E	HE
Ems						
Ems	r	EMS1	31	53°24′N	07°16′E	NI
		EMS2	31	53°24′N	07°16′E	NI
Eider						
Eider	r	EID1	31	54°19′N	09°09′E	SH
		EID2	14	54°18′N	09°15′E	SH
		EID3	12	54°20′N	09°10′E	SH

r, river; l, lake; c, canal; e, estuary; BB, Brandenburg; BW, Baden-Württemberg; BY, Bavaria; HE, Hesse; MV, Mecklenburg-Vorpommern; NI, Lower Saxony; NW, North Rhine-Westphalia; RP, Rhineland-Palatinate; SH, Schleswig-Holstein; SN, Saxony.

### Genetic marker analysis

Nine polymorphic microsatellites developed by Kohlmann and Kersten (2008) as well as the mitochondrial *cyt b* sequence were investigated as genetic markers for population genetic analysis of zander. A subset of 1057 samples was used in the microsatellite analysis with an average sample size of 22.0 ± 11.1 (mean ± SD; range = 1–52) individuals per site and 151.0 ± 140.0 (range = 31–433) individuals per river catchment. Microsatellites were amplified with a Mastercycle Gradient machine (Eppendorf, Hamburg, Germany) using the Qiagen® Multiplex PCR Kit (Qiagen, Hilden, Germany). Microsatellites MSL1–3, MSL4–6 and MSL7–9 were co-amplified, respectively, in multiplex PCR with 1.5 μL genomic DNA extract following the manufacturer's instruction, using reduced reaction volumes of 12.5 μL. Forward primers were 5′-labelled with fluorescence dyes D2, D3 and D4 (Sigma-Aldrich Biotechnology, Munich, Germany). The cycling program started with 15 min at 95°C, followed by 35 cycles of 0.5 min at 94°C, 1.5 min at 57°C, 1.5 min at 72°C, and finished with 10 min at 72°C. Fragment analysis was performed with a CEQ 8000 machine (Beckmann Coulter, Krefeld, Germany) equipped with an eight capillary injection system. Chromatograms were evaluated with the appending software package GenomeLab GeXP Genetic Analysis System version 10.2 by applying the Fragment Analysis Module.

A subset of 387 samples was used in the *cyt b* analysis with an average sample size of 7.1 ± 5.1 samples per site (range = 1–20). *Cyt b* sequences were amplified with a Mastercycle Gradient machine (Eppendorf, Hamburg, Germany) using a Maxima Hot Start PCR Master Mix (Fermentas GmbH, St. Leon-Rot, Germany) and zcyt-1F (5′-taatggcaagcctccgaaa-3′) and zcyt-2R (5′-ctgagctactaatgcaggatca-3′) as forward and reverse primers, respectively. PCRs were performed according to the manufacturer's instruction, using reduced reaction volumes of 20 μL. The cycling programme started with 4 min at 95 °C, followed by 35 cycles of 0.5 min at 95°C, 1.0 min at 51°C, 2.0 min at 72°C, finishing with 15 min at 72°C. Restriction analysis of the *cyt b* PCR fragment targeted the polymorphism described by Kohlmann et al. (2013) and was performed with enzyme Alw26I (Fermentas GmbH, St. Leon-Rot, Germany) according to the manufacturer's instruction using 10 μL of PCR product in a reduced reaction volume of 15 μL in 96-well plates. Electrophoresis was conducted with standard agarose gels. Restriction patterns were validated by double-strand sequencing of the *cyt b* PCR fragments of 168 samples used in the restriction analysis (average sample size = 3.4 ± 1.1 samples per site; range = 1–5). These analyses were completed by a sequencing service (Fraunhofer Institute for Molecular Biology and Applied Ecology, Aachen, Germany) using the above mentioned primers and additionally zcyt-1R (5′-gtttaagccaagggggttgt-3′) and zcyt-2F (5′-ctcgattctttgccttccac-3′) to obtain two overlapping fragments.

### Data analysis

Presence of null alleles and large allele drop-outs were tested with MICROCHECKER 2.2.3 (Van Oosterhout et al. 2004). Linkage disequilibrium was tested for 1476 loci combinations with Bonferroni corrections in all zander populations using GENEPOP 4.2 (Raymond and Rousset 1995). The same software was applied to test for Hardy-Weinberg deviations. Markov chain parameters were set at 10 000 dememorizations, 20 batches and 5000 iterations per batch.

Using microsatellite data, the genetic distance D_A_ (Nei et al. 1983), the chord distance D_C_ (Cavalli-Sforza and Edwards 1967) and the proportion-of-shared-alleles (Bowcock et al. 1994) were calculated as estimators of genetic distance with MSA 4.05. D_C_ was also calculated for individuals to determine whether populations with low sample size could be pooled (e.g. Danube and its tributary rivers). The Neighbour Joining algorithm implemented in MEGA 5 (Tamura et al. 2011) was used to calculate population trees based on all types of genetic distance data as well as one individual tree based on D_C_ distances. Bootstrapping of 10 000 replicates was performed with Phylip 3.6 (Felsenstein 1989) using the modules NEIGHBOUR and CONSENSE.

A subset of 387 zander samples were characterized by restriction analysis. Kohlmann et al. (2013) described two main haplotypes A and B for zander across Europe. These can be distinguished by cutting the *cyt* b PCR fragment with the restriction enzyme Alw26I, which yields two different restriction patterns. Based on these patterns, we calculated the share of each haplotype in each of the seven catchments.

To refine the results obtained with restriction analysis, a subset of 168 samples were sequenced and analysed with the software NETWORK 4.6.1.1 (Fluxus Technology Ltd, Suffolk, UK) to identify mutation patterns and possibly new haplotypes. Sequences from this study, together with known sequences of zander from different European regions, were used to construct a phylogenetic tree using the Neighbour Joining algorithm implemented in the software package MEGA 5. Sequences of the Volga zander *Sander volgensis* and perch *Perca fluviatilis* were included as outgroups.

To analyse and compare the genetic diversity across catchments, amova analyses were conducted with Arlequin 3.5.1.2 (Excoffier and Lischer 2010). Groups were defined according to river catchments, with Danube, Elbe and Oder belonging to the native distribution area, and Rhine, Weser, Ems and possibly Eider belonging to the invaded areas. All catchments comprised the main stream and its connected water bodies. To compare diversity levels of native with invaded ranges, two amovas were performed. The first run included and the second run excluded the invaded areas. Fixation indices and P values were obtained by significance testing with 1000 permutations.

Number of alleles (A_N_), allelic richness (A_R_) and heterozygosity (H_E_, H_O_, H_S_) were calculated with MSA 4.05 (Dieringer and Schlötterer 2003) and FSTAT 2.9.3.2 (Goudet 1995). The genetic diversity of populations of the native areas and of populations of the invaded areas was compared based on A_R_ and H_S_ obtained with FSTAT applying the statistical procedure described by Bronnenhuber et al. (2011). Accordingly, we first calculated mean A_R_ and H_S_ and 95% confidence intervals (CI) for all native populations together (*N* = 25). Next we tested for differences in genetic diversity (A_R_ and H_S_ independently) between the populations of the invaded and native areas by identifying values of the populations of the invaded areas that lay outside of the 95% confidence limits generated by the populations of the native areas. If more than one site was sampled in a river or lake in the invaded areas, the mean values were calculated for the respective water body and employed for comparison. Values of the populations of the invaded areas outside of the 95% CI of the native populations' values would be considered significantly different based on a 5% error threshold. Additionally, to further test for significant differences t-tests comparing the average A_R_ and H_S_ between the populations of the native (*N* = 25) and the non-native areas (*N* = 15) were conducted. (this test was not applicable to populations of single rivers and lakes due to the low number of sampling sites (*N* = 1–4) per water body).

STRUCTURE 2.3.2 (Falush et al. 2003) was used to infer the most likely population structure based on microsatellite data of 41 zander populations. The calculation was done with an admixture model without *a priori* population information, using a burn-in period of 30 000 and 20 000 subsequent MCMC repeats for each k value between one and six. The most likely number of ancestral populations was identified as the k value, where the change of likelihood dropped considerably and subsequent values plateaued (Δk criterion). Admixture of populations was calculated such that all individuals were assigned to each of the identified ancestral gene pools. Afterwards, their respective proportions of membership were computed. Values of populations within a catchment were partly pooled and plotted as pie-charts on a map of Germany to illustrate genetic admixture and distribution in a geographic context.

## Results

### Genetic structure as inferred from microsatellites

All microsatellites employed were polymorphic with an average number of 5.7 ± 1.4 alleles (mean ± SD) and an allelic richness of 4.5 ± 0.7 over all loci and populations (Table S1). All heterozygosity measures appeared grossly similar: H_O_ = 0.61 ± 0.07 (mean ± SD), H_E_ = 0.62 ±0.07 and H_S_ = 0.61 ± 0.07 over all loci and populations (Table S1).With respect to allelic distribution and inheritance over all loci and all populations (Table S2) deviation from Hardy–Weinberg equilibrium was found in 8.7% of all cases. About 60% of these correlated with significant heterozygote deficiency and potential presence of null alleles, which was detected in 2.2% of all cases. Large allele drop-out was not obvious. Linkage disequilibrium was detected in 1.7% of all possible allele combinations.

**Figure 1:**
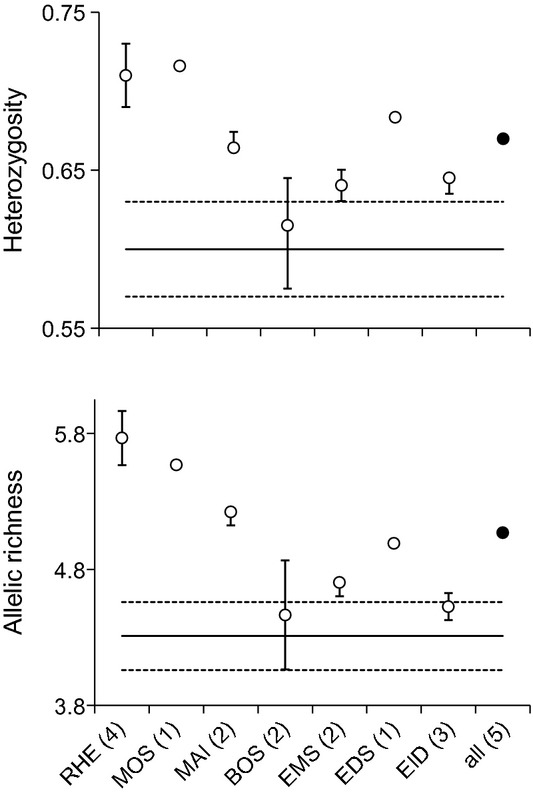
Heterozygosity (H_S_) and allelic richness (A_R_) calculated as estimators of genetic diversity in native and invaded areas. Diversity was increased in populations of the invaded range. – Solid line = mean calculated from all populations of the native areas (*n* = 25); dashed lines = 95% confidence interval. Populations of the invaded areas were pooled per river or lake and additionally over all sampling sites (number of pooled populations is given in brackets). Circles represent the mean and error bars the range of the values for pooled sampling sites (for clarity the error bar was omitted for data point ‘all’). Rhine catchment: RHE = RHE1–4, MOS, MAI = MAI3 + 4, BOS = BOS2 + 3; Ems catchment: EMS = EMS1 + 2; Weser catchment: EDS; Eider catchment: EID = EID1-3 (IDs are explained in Table 1).

For a more detailed analysis of genetic diversity, allelic richness (A_R_) and heterozygosity (H_S_) were chosen as estimators. When populations of single water bodies of the invaded areas (mean values calculated from one to four sampling sites per water body) were compared with the populations of the native areas (mean value of 25 sampling sites), they exhibited higher values in all cases. In most cases, these lay outside the 95% confidence interval (CI) of the native populations, and thus were considered significantly different (Fig. 1). Only A_R_ and H_S_ of the populations of Lake Constance, as well as H_S_ of Ems and A_R_ of Eider populations exhibited overlap with the CI, and thus were regarded as not significantly different (Fig. 1). Averaging all populations of the invaded area (mean values of 15 sampling sites) resulted in higher values of both estimators (A_R_ = 5.02 ± 0.57 (± 0.25); H_S_ = 0.67 ± 0.04 (±0.03); mean ± SD (± CI); indicated with data points ‘all’ in Fig. 1) as compared to populations of the native ranges (A_R_ = 4.26 ± 0.64 (± 0.25); H_S_ = 0.60 ± 0.07 (±0.03); mean ± SD (± CI); indicated with solid lines in Fig. 1). Upon removal of one of the Lake Constance populations (BOS2), which proved to be an outlier (Nalimov test), these differences were highly significant (*P* < 0.001) when tested with a one-sided Student′s *t*-test.

**Figure 2:**
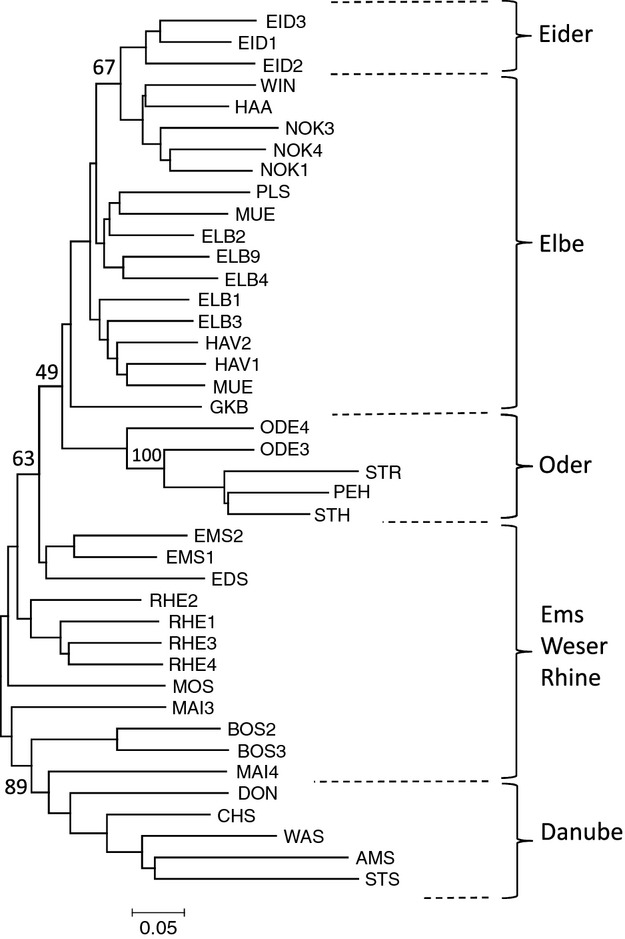
Neighbour joining tree of 41 zander populations based on chord distances. Populations of native areas showed catchment specific clustering and the populations of the invasion range linked the Elbe-Oder with the Danube clade. – Sample IDs are explained in Table 1. Numbers on branches indicate bootstrap support.

Of the three measures used to estimate genetic distances – D_A_, D_C_ and proportion-of-shared-alleles – D_C_ received the highest bootstrap support in Neighbour Joining (NJ) trees (Fig. 2). Because of low sample sizes (mostly < 5 samples per site) all river samples of the Danube catchment were pooled into one group designated DON (see Table 1). This was justified by individual distance calculations, in which individuals of different sampling sites grouped closely together (data not shown). As the lake populations (except Starnberger See) of the Danube catchments comprised sufficiently high sample numbers, these were kept as distinct populations. The NJ tree based on D_C_ values displayed a clear genetic structure with zander populations of the native areas forming distinct clades, receiving medium (63%, Elbe-Oder clade) to high (89%, Danube clade) bootstrap support. The Elbe-Oder group was further resolved into Elbe and Oder clades, but with rather low bootstrap support (49%). The Oder group formed a cluster together with, but clearly distinguished from populations of the brackish Baltic Sea populations (100% bootstrap support). The Elbe group split into two subgroups, one of which formed the sister group to the Eider/Nord-Ostsee-Kanal (NOK) clade. Within this clade, the brackish populations (NOK and adjacent waters) clearly separated as a sister group, as indicated by a moderately high bootstrap value (67%).

The populations of the invaded areas separated the Danube clade from the Elbe-Oder clade. More precisely, the river Main and Lake Constance populations (both belonging to the Rhine catchment) appeared more closely related to the Danube clade, while the other Rhine populations (Rhine itself and Mosel), together with the reservoir Edersee (Weser catchment) and the Ems populations, appeared genetically closer to the Elbe-Oder group. Most zander populations of the invaded areas formed clusters as well, for example, Ems, Rhine and Lake Constance populations, though with very low bootstrap support.

amova with all seven catchments (defined as groups) showed that most genetic variability was present on the population level (WPV in Table 2). Leaving out the groups of the invaded areas (Rhine, Weser and Ems) in the second amova resulted in a decrease of within-population variability from 88.6% to 85.5%. The concomitant increase of the genetic variability among catchments (AGV) by about 31% clearly indicated a higher genetic diversity in the populations of the invaded compared with the native ranges (increase from 7.3% to 10.6%). Variation among populations (APV) within a catchment remained almost the same (4.1% vs 3.9%) in both amovas.

**Table 2:** Genetic variability tested with amova. Within population variability was increased in zander populations outside the native ranges

Source of variation	d. f.	Sum of square	Variance components	Percent of variation	Fixation indices	*P* value*
All catchments						
AGV	6	428.6	0.232	7.27	0.073 (*F*_CT_)	<0.001
APV	34	321.5	0.131	4.13	0.044 (*F*_SC_)	<0.001
WPV	2073	5847.6	2.821	88.60	0.114 (*F*_ST_)	<0.001
Total	2113	6597.8	3.184	100	–	–
Without Rhine, Ems and Weser catchments						
AGV	3	323.2	0.338	10.58	0.106 (F_CT_)	<0.001
APV	25	227.0	0.126	3.94	0.044 (F_SC_)	<0.001
WPV	1449	3957.8	2.731	85.48	0.145 (F_ST_)	<0.001
Total	1477	4508.0	3.195	100	–	–

AGV, among group variation; APV, among population variation, WPV, within population variation.

* *P* values obtained by significance test with 1.000 permutations.

**Figure 3:**
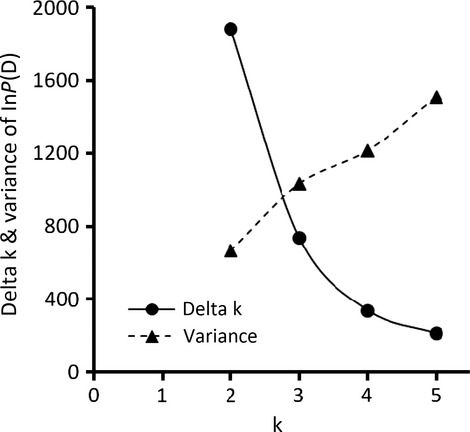
Admixture analysis revealed three genetic clusters as the most likely number, as indicated by a decrease in Δk and an increase in variance of calculated probabilities *P*(D).

Admixture analysis with STRUCTURE suggested a k value of three as the most likely number of existing clusters based on the minimal Δk criterion (Fig. 3). Each of these clusters was most frequent in either Danube or Elbe or Oder, suggesting these catchments as the most likely ancestral distribution areas of zander in Germany (Table 3). This was in agreement with the groupings as inferred from distance tree based analysis (Fig. 2) and as employed in the definition of the groups (i.e. catchment-specific) in the amovas (Table 2). The best fit model from the STRUCTURE analysis suggested contributions of all ancestral clusters to all sampling sites, albeit to different extents.

**Table 3:** Admixture analysis revealed three genetic clusters of zander populations belonging to the native catchments of Danube, Elbe and Oder, respectively

			Proportion of ancestry:		
			
Drainage system	Sample ID*	No. in Fig. 4†	Danube	Elbe	Oder
Danube	DON	1	0.878	0.070	0.053
	CHS	1.1	0.823	0.116	0.061
	WAS	1.1	0.864	0.079	0.057
	AMS	1.2	0.941	0.037	0.022
	STS	1.2	0.943	0.036	0.021
Elbe	ELB1	2	0.058	0.715	0.227
	ELB2	2	0.118	0.713	0.168
	ELB3	2	0.091	0.633	0.276
	ELB4	2	0.229	0.652	0.119
	ELB9	2	0.210	0.653	0.138
	NOK1	2.1	0.052	0.834	0.114
	NOK3	2.1	0.039	0.848	0.113
	NOK4	2.1	0.041	0.852	0.107
	HAA	2.1	0.034	0.868	0.098
	WIN	2.1	0.059	0.869	0.071
	HAV1	2.2	0.081	0.522	0.397
	HAV2	2.2	0.052	0.663	0.285
	MUE	2.3	0.097	0.451	0.452
	GKB	2.3	0.185	0.501	0.314
	MUR	2.4	0.289	0.615	0.096
	PLS	2.4	0.179	0.748	0.073
Oder	ODE3	3	0.076	0.133	0.791
	ODE4	3	0.155	0.139	0.706
	PEH	3.1	0.032	0.056	0.912
	STH	3.1	0.040	0.064	0.896
	STR	3.1	0.021	0.049	0.930
Rhine	RHE1	4	0.438	0.200	0.362
	RHE2	4	0.488	0.296	0.217
	RHE3	4	0.470	0.233	0.297
	RHE4	4	0.394	0.208	0.398
	MOS	4.1	0.570	0.345	0.085
	MAI3	4.2	0.490	0.452	0.058
	MAI4	4.2	0.784	0.122	0.094
	BOS2	4.3	0.640	0.120	0.240
	BOS3	4.3	0.772	0.097	0.132
Weser	EDS	5	0.378	0.207	0.415
Ems	EMS1	6	0.314	0.433	0.253
	EMS2	6	0.441	0.356	0.202
Eider	EID1	7	0.241	0.648	0.110
	EID2	7	0.251	0.571	0.178
	EID3	7	0.256	0.657	0.086

NOK, Nord-Ostsee-Kanal.

Shaded areas indicate proportions of native ancestries.

* See Table 1 for definition of sample ID.

**Figure 4:**
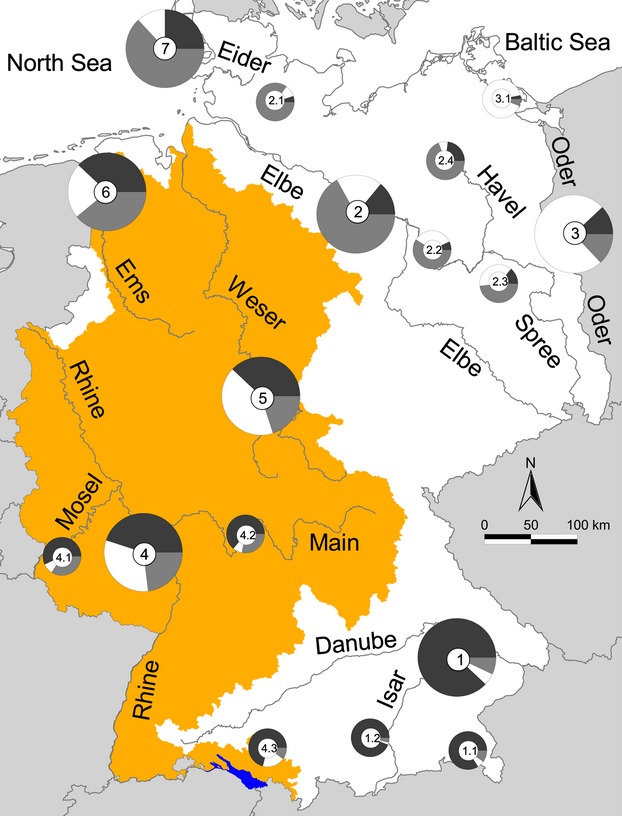
Genetic admixture of zander populations in German inland and coastal waters. Levels of admixture were higher in the invasion ranges (colored area) as compared with the native ranges. The big lake (colored blue) at the southern borderline of Germany is Lake Constance. Black, grey and white colors in the pie charts indicate the genetic proportion of Danube, Elbe and Oder ancestry respectively. Numbers indicate pooled populations displayed in Table 3.

† Samples with the same number have been pooled for a clear presentation in Fig. 4.

The populations of the native areas showed low to medium levels of admixture. Their genetic composition was dominated by ancestral gene pools, which had a share of 88%, 75% and 67% in Danube, Oder and Elbe, respectively (pie charts 1, 3 and 2, respectively, in Fig. 4; note: all subsequently indicated numbers in brackets of this paragraph refer to pie chart numbers in Fig. 4). Within these catchments, even higher levels of native ancestry were detected: all large alpine lakes (belonging to the Danube catchment) were dominated by genes of Danube ancestry, with levels of >90% in Ammersee and Starnberger See (1.2) and >80% in Chiemsee and Waginger See (1.1). With 85%, the highest proportion of Elbe ancestry was detected in the Nord-Ostsee-Kanal (2.1), a brackish environment connecting the North Sea with the Baltic Sea. Ninety-one percent of the ancestry in the brackish Oder estuary and adjacent Baltic Sea area was from the Oder (3.1). Ancestry coefficients as inferred with STRUCTURE suggested that overall levels of admixture were more pronounced in the populations outside the native range with grossly comparable proportions of each of the three gene pools. In the Rhine catchment (4), the distribution was 45% Danube (D), 32% Oder (O) and 23% Elbe (E); in the Ems catchment (6), it was 38% D, 23% O and 39% E; and in the Weser catchment (5), it was 38% D, 42% O and 21% E. Within the Rhine catchment, Lake Constance (4.3), with 71%, and the large tributaries Main (4.2) and Mosel (4.1), with 64% and 57%, respectively, exhibited elevated Danube ancestry. The river Eider (7) in the very north of Germany is a catchment on its own and was most likely invaded by zander populations from the river Elbe, to which it is connected via the Nord-Ostsee-Kanal. 63% Elbe ancestry appeared as a strong indication of this fact.

Lake and river populations exhibited no consistent patterns in their degrees of admixture, that is, there was not a clear tendency of more or less admixture in riverine or lacustrine habitats. For example, within the Danube catchment, zander populations of Ammersee and Starnberger See (pie chart 1.2 in Fig. 4; note: all subsequently indicated numbers in brackets of this paragraph refer to pie chart numbers in Fig. 4) were less admixed than the respective river populations (1), but Chiemsee and Waginger See (1.1) showed comparable or higher levels of admixture. Similarly, within the Elbe catchment lake populations of Müritz and Plauer See (2.4) showed comparable levels of admixture to the respective river populations (2), but populations of the Spree lakes Müggelsee and Großer Kossenblatter See (2.3) were much more admixed. Outside the native range of zander, Lake Constance populations (4.3) had a lower level of admixture compared with Rhine populations (4), but this was also true for the river populations of Main (4.2) and Mosel (4.1) belonging to the same catchment.

### Haplotype analysis based on the mitochondrial *cyt b* gene

**Figure 5:**
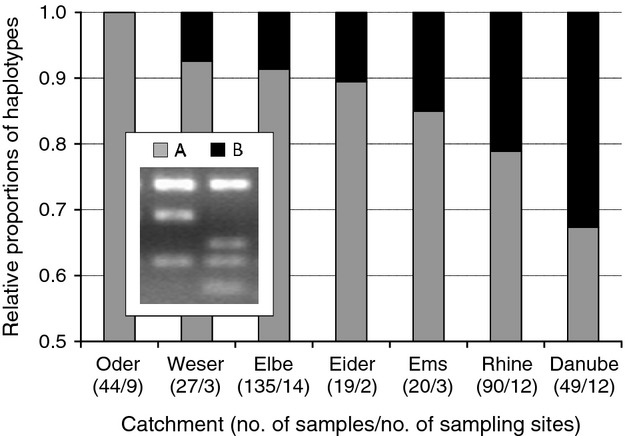
Relative proportions of haplotypes A and B per river catchment (columns) identified by restriction analysis. Haplotype B was dominant in the Danube catchment and spread particularly towards the invasion ranges. – Haplotype B bore an additional restriction site for enzyme Alw26I depicted in the legend. Numbers in brackets below catchment denotations specify total sample size and number of sampling sites.

Restriction analysis of the mitochondrial *cyt b* gene differentiated two haplotypic lineages A and B of zander described previously by Kohlmann et al. (2013). Haplotype A was the most frequent type found in all catchments with proportions of at least 65% (Fig. 5). Haplotype B appeared most frequent in the Danube catchment (> 30%), but was also present with high shares in the catchments of Rhine (about 20%) and Ems (about 15%). Proportions of this haplotype were less in Weser, Eider and Elbe (about 10% each), and it was completely absent in the Oder samples.

NETWORK analysis of mitochondrial *cyt b* sequences confirmed the two main haplotypic lineages A and B identified by restriction analysis (Figure S1). Both included two subtypes, of which only A1 was found in higher numbers, while all others were rare (one or two individuals). All but one subtype were separated by a single mutation from the respective main type. B2 present in two samples from Ammersee (Danube catchment) was a notable exception, because it was separated by two mutational steps from B and by three mutations from A, and thus represented an intermediate haplotype.

A representative sequence of each haplotype was deposited in Genbank (Acc. No. KC960516-KC960521) and compared with sequences of zander from geographic regions outside Germany. Neighbour Joining analysis showed that no haplotypes other than A and B have been identified for Europe to date. All sequences grouped with one of these two main types, which represented sister clades (Figure S2). Moreover, both clades were significantly different from the Volga zander – a close relative of zander – and perch, which were employed as outgroups.

## Discussion

We investigated the contemporary invasive spreading of zander in German inland waters, and analysed how this was possibly driven by admixture of different genetic lineages after multiple human-assisted secondary contacts. Analysis of nine species-specific microsatellites and the mitochondrial *cyt b* gene revealed conspicuous asymmetric distribution of admixed genetic ancestries indicating gene flow predominantly from the Danube, Elbe and Oder towards Rhine, Weser and Ems catchments. We discuss the population differentiation of zander as a result of different initial situations in the native versus the newly colonized ranges.

### Contemporary genetic structure & diversity

Microsatellite based genetic distance analysis identified the populations of the Danube, Oder and Elbe catchments as three distinct clades, indicating a divergent development of these populations after postglacial re-colonization of the native areas (Kottelat and Freyhof 2007). By contrast, the populations in the areas where zander was introduced in the late 19th century (Lehmann 1931; Welcomme 1988; Schmidt 1993; Löffler 1998) showed less structuring and a positioning between the Danube and the Elbe-Oder clades. We interpreted these patterns as evidence of recent admixture caused by human-assisted secondary contact of the native genetic lineages resulting in intermediate genotypes of zander populations in the invaded areas.

In contrast to the microsatellite data, sequence analysis of the mitochondrial *cyt b* gene resolved only two genetic lineages. This is in agreement with Kohlmann et al. (2013), who reported the existence of two haplotypes, A and B, for zander in Europe, most likely as a consequence of bottlenecks during glaciation and subsequent founder effects (Haponski and Stepien 2013). Phylogenetic and network analysis displayed the two lineages as clearly separated sister clades corresponding to either the A or the B type or their subtypes. Whereas the A type was dominant in all drainages, the B type was more specific to the Danube catchment and most likely originated there. This was further supported by elevated proportions of the B haplotype in the Danube drainage. More evidence was provided by subtype B2, which was detected two times in a lake (Ammersee) within the Danube catchment: while all other subtypes differed by only one mutation from their respective main type, B2 was different by three mutations from haplotype A and by two mutations from haplotype B. The most likely explanation is that B2 was derived from haplotype A and presents a precursor of haplotype B, which arose specifically in the Danube catchment. This would support Kohlmann et al. (2013) as well as Haponski and Stepien (2013) speculating a south-eastern European origin of zander in the Ponto-Caspian region. Thus, based on nuclear and mitochondrial genetic markers, two evolutionarily significant units can be justified for zander in Germany, comprising the Danube populations on one hand and the Elbe-Oder populations on the other. Danubian lineages have been demonstrated for a range of European freshwater fish such as barbel *Barbus barbus*, bullhead *Cottus gobio*, chub *Leuciscus cephalus*, grayling *Thymallus thymallus*, perch (all species summarized in Seifertová et al. 2012) and brown trout *Salmo trutta* (Bernatchez et al. 1992). These findings suggest that the Danube has been an important southern refuge during glacial periods from which re-colonization and subsequent development of distinct genetic lineages was possible.

Divergent genetic lineages may come into secondary contact through natural events or human activity after long periods of geographic isolation, which becomes obvious by increased diversity. In fish, this has been observed for sculpins (Nolte et al. 2006), round goby *Neogobius melanostomus* (Bronnenhuber et al. 2011) and sticklebacks (Lucek et al. 2010). In the current study, zander diversity obviously increased as there was a marked increase in within-population variability in the invaded ranges, and elevated levels of allelic richness and heterozygosity as compared with the populations in the native areas. The non-native zander population of Lake Constance, however, was an exception by showing comparable diversity to zander populations of the native range. This might be due to the genetic influence of the Danube, which served as a source for introducing zander in the late 19th century and later on for repeated stocking activities (Lehmann 1931; Welcomme 1988; Schmidt 1993; Löffler 1998), and would thus represent a sustainable genetic effect of a founder population (Waters et al. 2013). Thaulow et al. (2013) recently provided another example in fish by showing that lake-spawning brown trout populations introduced 45 years ago into a lake in Norway are still more similar to the founder population than to populations used subsequently for stocking. In addition to successful introduction, their case study demonstrates how established founder populations effectively outcompete later arriving populations, a phenomenon termed ‘competitive exclusion’ by Waters (2011). The lower genetic diversity of zander in Lake Constance might be a result of this mechanism as well, reflecting genetic stabilization, which was determined by mutation and drift rather than gene flow (Björklund et al. 2007). By contrast, when founder populations with different genetic backgrounds arrive simultaneously in an environment without adapted competitors, these have equal opportunity to establish or may even benefit from admixture (Nolte et al. 2005; Lucek et al. 2010; Bronnenhuber et al. 2011) explaining the greater levels of genetic diversity of zander populations in the more recently colonized rivers and lakes of the invaded areas.

### Asymmetric admixture patterns & gene flow

We identified three ancestral genetic lineages with microsatellite based admixture analysis, which unambiguously coincided with the three clades harboured in the native river catchments of Danube, Oder and Elbe. Although zander populations of each of these areas exhibited signs of admixture with other genetic lineages, indicating that introgression has happened, this was much more pronounced in the non-native catchments of Rhine, Weser and Ems. Populations in these river systems were completely admixed and exhibited more or less balanced proportions of each of the three genetic lineages, meaning that fish from all native catchments must have been translocated or migrated into each of the rivers in the non-native range.

By contrast, much less influence of non-native genes was detected in the native range, suggesting effective buffering via competitive exclusion (Waters 2011) mediated either by demographic (high-density blocking) or genetic (local adaptation) factors or both. In salmonids, for example, the fitness advantage of local adaptation has been clearly identified in a recent meta-analysis, and has been shown to increase with genetic distance (Fraser et al. 2011). Similarly Nolte et al. (2006) demonstrated that an invasive sculpin lineage was inferior to a locally adapted type. For the same reason, it is possible that native zander populations, due to their long-lasting evolutionary history, were competitively superior to newly arriving conspecifics with a different genetic background. Moreover, stocked fish might be mal-adapted to natural environments due to the fact that they or their parental generations spent part of their life-cycle in a hatchery (Araki et al. 2007; Christie et al. 2012). There are several examples in the literature indicating that hatchery rearing of fish results in a considerable loss of genetic diversity and fitness (see Araki and Schmid 2010 for a comprehensive review). All these factors can contribute to competitive exclusion mechanisms and explain the observed limited introgression of foreign genes into ancestral gene pools despite open transfer routes via canals and on-going stocking activity.

Within the groups of native zander, the brackish populations of the Oder (Oder estuary and coastal Baltic Sea area) and Elbe catchments (Nord-Ostsee-Kanal and adjacent waters) exhibited particularly high proportions of native gene ancestry. Living in higher salinity waters and spawning in freshwater tributaries requires genetic adaptation, which may not be acquired *ad hoc*, and which may lead to genetically differentiated populations that exhibit greater fitness in brackish waters compared with zander exclusively adapted to freshwater environments. Säisä et al. (2010) found pronounced genetic differentiation between zander populations from coastal areas and freshwater lakes in Finland (*F*_ST_ ≥ 0.25) that supports this idea. However, in our own study, we observed only small genetic differences between German brackish and freshwater populations (data not shown). Larsen et al. (2005) investigated brackish pike *Esox lucius* populations in Denmark and observed very low introgression rates (<1%) despite intensive stocking with freshwater pike over more than four decades. Local adaptation might be an important reason for their findings, and might also be relevant in brackish zander populations.

Also the freshwater populations of the Danube catchment exhibited pronounced levels of native genetic ancestry. A more effective geographical isolation as compared with other catchments might be a reason here, protecting the local gene pool against foreign genetic influences. However, the Danube was likely the source of haplotype B of the mitochondrial *cyt b* gene, which spread towards the other catchments, where generally the A haplotype dominated. Intriguingly, much like it was observed with microsatellite markers, gene flow seemed to spread foremost to the invaded areas, while the native areas appeared less (Elbe) or not at all admixed (Oder).

### An alternative explanation and concluding remarks

As an alternative cause for the observed asymmetric admixture patterns, unequal levels of stocking in the native versus the newly colonized areas might be considered. Unfortunately, we could not investigate this hypothesis further due to the lack of a comprehensive documentation on stocking activities in Germany. However, more and more evidence is accumulating that stocking in the presence of self-sustaining fish stocks is rarely successful in a range of freshwater fish species (vendace *Coregonus albula* in Mehner et al. 2010; pike in Larsen et al. 2005; charr *Salvelinus alpinus* in Englbrecht et al. 2002; trout in Hansen 2002). Strong evidence, that this also holds true for zander, is provided in a recent work of Salminen et al. (2012). Studying three lakes, they found low replacement of native gene pools, when natural recruitment of zander was high, but they observed extended replacement and high levels of admixture, when self-recruitment was low. From this point of view, the asymmetric admixture patterns observed in our study are best explained as the outcome of prevailing natural selective forces and demographic determinants rather than being due to unequal stocking. Thus, in the native areas, competitive exclusion very likely prevents extensive introgression and preserves ancestral gene pools (Waters 2011), whereas in the areas outside the native range admixture is advantageous by allowing rapid adaptation and effective invasion of new habitats (Abbott et al. 2013). The exact nature of these adaptations, however, cannot be identified with neutral genetic markers, and remains to be investigated by future studies. Nevertheless, an implication of our study is that further stocking should take notice of the genetic structure of zander and confine stocking material to the particular genetic lineages already present in that area.

## Data archiving statement

Microsatellite raw data (genotypes) for this study are available from the Dryad Digital Repository: http://dx.doi.org/10.5061/dryad.3180v.

## Supporting Information

**Figure S1.** Network analysis of *cyt b* sequences revealed two main haplotypes A and B. Each of these comprised two subtypes with B2 representing an intermediate of the two main types. – Circle sizes are proportional to the number of individuals detected with a specific haplotype. The number of mutation steps separating different haplotypes is indicated by black dots.

**Figure S2.** Neighbour joining tree based on mitochondrial *cyt b* sequences demonstrating the dimorphic nature of the mitochondrial gene in Europe. – A, A1, A2, B, B1, B2 = haplotypes identified in the current study; Accession numbers and country codes = haplotypes isolated by others from different European sites; numbers specify bootstrap values ≥70%; Volga zander and perch are included as outgroups.

**Table S1.** Microsatellite polymorphism and genetic diversity. A_R_ and H_S_ (shaded) have been employed for calculations depicted in Fig. 1.

**Table S2.** Tests for allelic distribution and inheritance.
