# Supplementary figures and images for: Population differentiation of zander (Sander lucioperca) across native and newly colonized ranges suggests increasing admixture in the course of an invasion
Source: Evol Appl. 2014 Apr 26;7(5):555–68. doi: 10.1111/eva.12155 (PMC4055177; doi:10.1111/eva.12155)

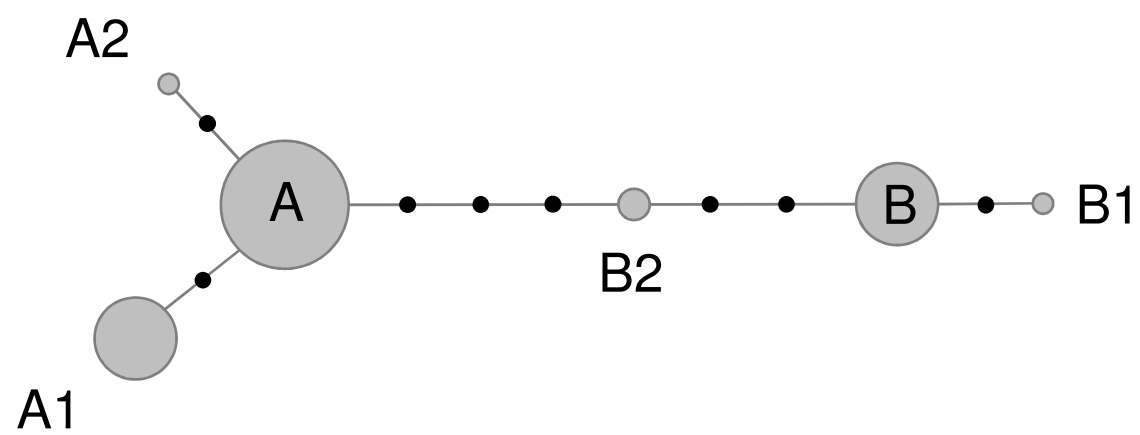

fig. S1

Supplement: Supplementary file 1 [file eva0007-0555-SD1.pdf]

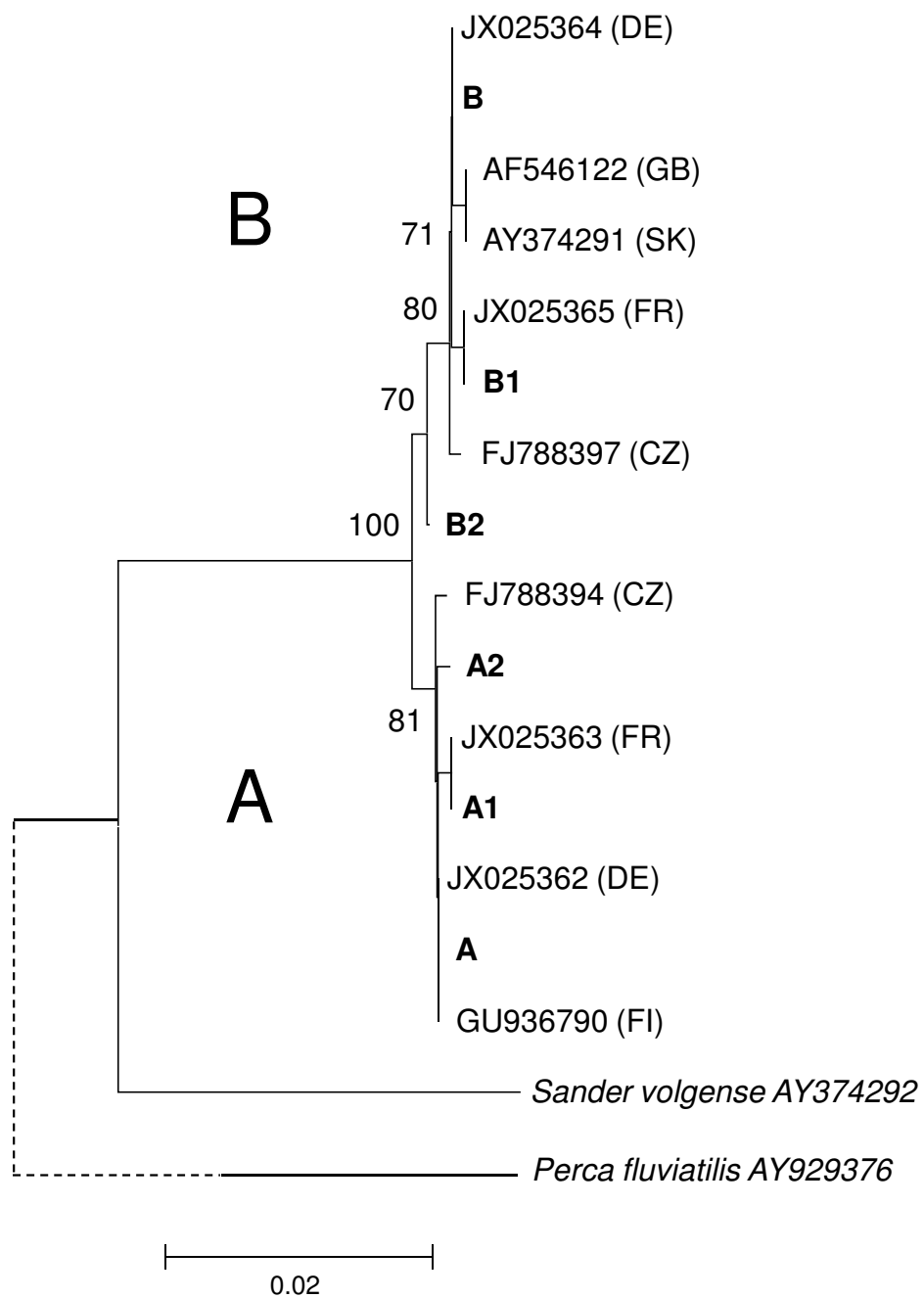

fig. S2

Supplement: Supplementary file 2 [file eva0007-0555-SD2.pdf]
