# Supplementary material for: Population differentiation of zander (Sander lucioperca) across native and newly colonized ranges suggests increasing admixture in the course of an invasion
Source: Evol Appl. 2014 Apr 26;7(5):555–68. doi: 10.1111/eva.12155 (PMC4055177; doi:10.1111/eva.12155)
Supplement: Supplementary file 3 [file eva0007-0555-SD3.doc]

**Table S1: Microsatellite polymorphism and genetic diversity. A_R_ and H_S_ (shaded) have been employed for calculations depicted in fig. 1.**

| **Drainage system** | **sample ID** | **A_N_** | **A_R_** | **H_S_** | **H_O_** | **H_E_** |
| --- | --- | --- | --- | --- | --- | --- |
|  |  |  |  |  |  |  |
| Danube | AMS | 6,1 | 4,4 | 0.57 | 0.56 | 0.57 |
|  | CHS | 5,1 | 4,2 | 0.62 | 0.60 | 0.62 |
|  | DON | 8,1 | 4,4 | 0.73 | 0.68 | 0.73 |
|  | STS | 2,4 | ND^1^ | 0.46 | 0.47 | 0.43 |
|  | WAS | 4,9 | 4,3 | 0.59 | 0.58 | 0.60 |
|  |  |  |  |  |  |  |
| Elbe | ELB1 | 5,0 | 4,2 | 0.59 | 0.57 | 0.60 |
|  | ELB2 | 5,2 | 4,3 | 0.65 | 0.68 | 0.66 |
|  | ELB3 | 5,2 | 4,1 | 0.58 | 0.60 | 0.58 |
|  | ELB4 | 6,8 | 4,8 | 0.66 | 0.62 | 0.67 |
|  | ELB9 | 6,1 | 4,6 | 0.66 | 0.60 | 0.67 |
|  | GKB | 4,8 | 3,9 | 0.60 | 0.61 | 0.60 |
|  | HAA | 4,1 | 3,4 | 0.54 | 0.54 | 0.54 |
|  | HAV1 | 5,6 | 4,4 | 0.61 | 0.59 | 0.62 |
|  | HAV2 | 5,0 | 4,0 | 0.57 | 0.54 | 0.57 |
|  | MUE | 5,4 | 4,2 | 0.61 | 0.64 | 0.61 |
|  | MUR | 5,0 | 4,3 | 0.68 | 0.63 | 0.69 |
|  | NOK1 | 3,8 | 3,4 | 0.51 | 0.52 | 0.52 |
|  | NOK3 | 3,3 | 3,3 | 0.55 | 0.48 | 0.50 |
|  | NOK4 | 4,2 | 3,5 | 0.53 | 0.54 | 0.54 |
|  | PLS | 4,9 | 4,2 | 0.67 | 0.64 | 0.68 |
|  | WIN | 4,1 | 3,5 | 0.50 | 0.48 | 0.51 |
|  |  |  |  |  |  |  |
| Oder | ODE3 | 8,0 | 5,2 | 0.59 | 0.59 | 0.59 |
|  | ODE4 | 7,4 | 5,8 | 0.67 | 0.69 | 0.68 |
|  | PEH | 7,0 | 4,7 | 0.55 | 0.58 | 0.56 |
|  | STH | 6,9 | 4,5 | 0.55 | 0.53 | 0.56 |
|  | STR | 4,2 | 3,7 | 0.48 | 0.49 | 0.48 |
|  |  |  |  |  |  |  |
| Rhine | BOS2 | 5,3 | 4,0 | 0.58 | 0.59 | 0.58 |
|  | BOS3 | 6,1 | 4,8 | 0.64 | 0.60 | 0.64 |
|  | MAI3 | 5,1 | 5,1 | 0.65 | 0.56 | 0.66 |
|  | MAI4 | 6,1 | 5,2 | 0.65 | 0.69 | 0.66 |
|  | MOS | 7,8 | 5,5 | 0.71 | 0.71 | 0.72 |
|  | RHE1 | 6,7 | 5,5 | 0.69 | 0.67 | 0.70 |
|  | RHE2 | 7,9 | 5,9 | 0.73 | 0.71 | 0.73 |
|  | RHE3 | 7,2 | 5,7 | 0.71 | 0.68 | 0.72 |
|  | RHE4 | 7,8 | 5,8 | 0.68 | 0.72 | 0.69 |
|  |  |  |  |  |  |  |
| Weser | EDS | 6,8 | 4,9 | 0.68 | 0.70 | 0.68 |
|  |  |  |  |  |  |  |
| Ems | EMS1 | 6,2 | 4,7 | 0.63 | 0.61 | 0.63 |
|  | EMS2 | 5,9 | 4,6 | 0.64 | 0.65 | 0.65 |
|  |  |  |  |  |  |  |
| Eider | EID1 | 5,4 | 4,4 | 0.64 | 0.63 | 0.64 |
|  | EID2 | 5,0 | 4,6 | 0.63 | 0.66 | 0.64 |
|  | EID3 | 4,7 | 4,5 | 0.64 | 0.64 | 0.65 |
| **all** | **mean** | **5.7** | **4.5** | **0.61** | **0.61** | **0.62** |
|  | **SD^2^** | **1.4** | **0.7** | **0.07** | **0.07** | **0.07** |

^1^ ND = not determined (sample size < 10). ^2^SD = standard deviation; A_N_ = total number of alleles, A_R_ = allelic richness, H_S_ = expected heterozygosity (FSTAT 2.9.3.2), H_E_ = expected and H_O_ = observed heterozygosity (MSA 4.05).
