# Supplementary material for: Population differentiation of zander (Sander lucioperca) across native and newly colonized ranges suggests increasing admixture in the course of an invasion
Source: Evol Appl. 2014 Apr 26;7(5):555–68. doi: 10.1111/eva.12155 (PMC4055177; doi:10.1111/eva.12155)
Supplement: Supplementary file 4 [file eva0007-0555-SD4.docx]

**Table S2**: Tests for allelic distribution and inheritance

**A**: Test for null alleles and large allel drop-out (Microchecker 2.2.3)

| **loci** | **Danube:** | **Elbe:** | | **Oder:** | **Rhine:** | | | **total no.** |
| --- | --- | --- | --- | --- | --- | --- | --- | --- |
|  | **CHS** | **MUR** | **ELB9** | **STH** | **MOS** | **MAI3** | **BOS2** |  |
| MSL1 |  |  |  |  | x |  |  | 1 |
| MSL3 |  | x |  | x |  |  |  | 2 |
| MSL4 | x |  | x |  |  | x |  | 3 |
| MSL7 |  |  |  |  |  |  | x | 1 |
| MSL8 |  |  |  |  |  | x |  | 1 |
| **total no.** | 1 | 1 | 1 | 1 | 1 | 2 | 1 | **8** |

**🡪 8 of 369 alleles = 2.17% null alleles (no large allele drop-out)**

**B**: Test for Hardy-Weinberg equilbrium (Genepop 4.2)

| **loci** | **Danube** | | **Elbe** | | | | | | | | **Oder** | | | **Rhine** | | | | **Weser** | **total no.** |
| --- | --- | --- | --- | --- | --- | --- | --- | --- | --- | --- | --- | --- | --- | --- | --- | --- | --- | --- | --- |
|  | **CHS** | **DON** | **ELB3** | **ELB4** | **ELB9** | **HAA** | **HAV2** | **MUR** | **NOK1** | **WIN** | **ODE3** | **ODE4** | **PEH** | **BOS2** | **BOS3** | **MAI3** | **RHE3** | **EDS** |  |
| MSL1 |  |  | x, o |  | x |  |  |  |  |  |  |  |  |  |  |  |  |  | 2 |
| MSL2 |  | x |  |  |  |  |  |  |  |  |  |  |  | x |  |  |  |  | 2 |
| MSL3 |  |  |  |  |  | x, o |  | x, o, + | x |  |  |  |  | x, o |  |  |  | x | 5 |
| MSL4 | x, o, + | x |  | x | x, o, + |  |  |  |  |  |  |  |  |  |  | x, o, + |  |  | 5 |
| MSL5 |  |  |  |  |  |  |  |  |  |  |  |  |  |  | x, o | x, o |  |  | 2 |
| MSL6 |  | x, o |  |  |  |  |  |  |  |  |  | x |  |  |  | x | x |  | 4 |
| MSL7 |  | x, o |  |  | x, o |  |  | x, o |  |  |  | x | x, o |  | x, o |  |  |  | 6 |
| MSL8 |  | x, o |  |  |  |  | x |  |  |  |  |  |  |  |  | x, o, + |  |  | 3 |
| MSL9 |  | x, o |  |  |  |  |  |  |  | x, o | x |  |  |  |  |  |  |  | 3 |
| **total no.** | 1 | 6 | 1 | 1 | 3 | 1 | 1 | 2 | 1 | 1 | 1 | 2 | 1 | 2 | 2 | 4 | 1 | 1 | **32** |

x = significant in HWE probability test, o = significant in HWE heterozygote deficit, + = null alleles possible

**🡪 32 of 369 alleles = 8.67% alleles not in HWE**

**C:** Test for linkage disequilbrium (Genepop4.2)

| **MSL loci pairs** | **Danube** | **Elbe** |  |  | **Eider** | **Ems** |  | **Rhine** |  |  |  | **total no.** |
| --- | --- | --- | --- | --- | --- | --- | --- | --- | --- | --- | --- | --- |
|  | **DON** | **HAA** | **MUE** | **WIN** | **EID1** | **EMS1** | **EMS2** | **RHE3** | **MOS** | **RHE2** | **MAI4** |  |
| 1x2 |  |  |  |  |  |  |  | x |  |  |  | 1 |
| 1x4 |  |  |  |  |  |  | x | x |  |  | x | 3 |
| 1x9 |  |  |  |  |  |  |  | x |  |  |  | 1 |
| 2x3 |  |  | x |  |  |  |  |  |  |  |  | 1 |
| 2x5 |  |  |  |  |  |  | x |  |  |  |  | 1 |
| 2x6 |  |  |  |  |  |  | x |  |  |  |  | 1 |
| 2x9 |  |  |  |  |  |  | x | x |  |  |  | 2 |
| 3x4 |  |  |  |  |  |  |  | x |  |  |  | 1 |
| 4x5 |  |  |  |  |  |  | x |  | x |  |  | 2 |
| 4x6 |  |  |  |  |  |  |  | x |  |  |  | 1 |
| 5x8 | x | x | x | x | x | x | x | x | x | x |  | 10 |
| 5x9 |  |  |  |  |  |  |  |  | x |  |  | 1 |
| **total no.** | 1 | 1 | 2 | 1 | 1 | 1 | 6 | 7 | 3 | 1 | 1 | **25** |

**🡪 25 of 1476 total loci combinations = 1.69% show linkage disequilibrium**
